# A Rare Case of Fatal *Balamuthia mandrillaris* Encephalitis in a Pediatric Patient: Diagnostic Challenges

**DOI:** 10.1155/crdi/5594656

**Published:** 2026-10-13

**Authors:** Isabella Amado, Yukta Italia, Rebecca Luke, Mary Suzanne Whitworth

**Affiliations:** ^1^ Anne Burnett Marion School of Medicine at TCU Fort Worth Texas USA; ^2^ Texas College of Osteopathic Medicine Fort Worth Texas USA texascollege.edu; ^3^ Department of Neurology Cook Children’s Medical Center Fort Worth Texas USA; ^4^ Department of Infectious Diseases Cook Children’s Medical Center Fort Worth Texas USA

## Abstract

*Balamuthia mandrillaris* is a rare and lethal amoeba that causes granulomatous amoebic encephalitis (GAE), a severe brain infection with a high mortality rate. This manuscript presents a case study of a 5‐year‐old female patient with *B. mandrillaris* amoebic encephalitis. She presented from an outside hospital (OSH) with daily fevers, fatigue, and worsening mental status. She was without comorbidities, and sick contacts were denied. Diagnosis was challenging due to nonspecific encephalitis symptoms and nonspecific neurological imaging. Neuroimaging revealed symmetric diffuse restriction in the medial aspect of the basal ganglia. Autoimmune antibody testing was unrevealing. Amoebic encephalitis was considered when exhaustive testing was negative. Treatment with antiamoebic agents was initiated while free‐living amoebic PCR panel was pending. The diagnosis was confirmed through PCR testing on cerebrospinal fluid (CSF), highlighting the importance of molecular techniques in diagnosing *B. mandrillaris* infections. After confirmation, treatment was updated per CDC recommendations. However, her condition continued to worsen, with progression of hemorrhage, ventriculomegaly, and ultimately herniation. Unfortunately, devastating neurological damage to the brainstem had already occurred, leading to brain death. Early recognition and accurate diagnosis are crucial for timely treatment and prevention of mortality by *B. mandrillaris* encephalitis. The application of molecular techniques offers promising advancements in this field.

## 1. Introduction

*Balamuthia mandrillaris* is a rare and lethal single‐cell amoeba that is found in soil and notably causes granulomatous amoebic encephalitis (GAE), a severe brain infection leading to death in most cases [1]. The first case of GAE caused by *B. mandrillaris* was reported in 1990, with only a limited number of cases documented worldwide [1]. Between 1974 and 2016, 109 cases of *Balamuthia* GAE were reported in the United States, with only 10 survivors, leading to a mortality rate of > 90% [2]. The Centers for Disease Control and Prevention (CDC) noted over 200 reported cases worldwide over the last 30 years, mostly in immunocompromised individuals, with a disproportionate involvement of Hispanic patients (55%) among US cases [3]. *Balamuthia* has been found in dust, soil, and water in many places around the world, with an increased incidence of *B. mandrillaris* GAE cases in the warmer climates of the Southwestern United States and Latin America [1]. It is not transmitted from person to person except through organ transplantation [4, 5]. Although historically rare, the incidence of GAE caused by *Balamuthia* is increasing, making it a significant public health concern.

## 2. Case Presentation

A previously healthy five‐year‐old female developed daily fevers (up to 102℉) and vomiting associated with fatigue and malaise. She was seen in an outside hospital (OSH) emergency department (ED) 10 days after symptom onset and discharged with a diagnosis of viral illness. She continued having the same symptoms and was taken back to the OSH ED 2 days later. A complete blood count (CBC) demonstrated mild leukopenia and lymphopenia with normal platelets. A complete metabolic panel (CMP) and procalcitonin were normal. A brain computerized tomography (CT) scan revealed possible ischemia or inflammation. Based on continued clinical symptoms and an abnormal CT scan, she was admitted to an OSH.

The following day, a brain magnetic resonance imaging (MRI) scan showed symmetric restricted diffusion involving the medial aspect of the basal ganglia. There was no enhancement. This was thought to be an ischemic process. A lumbar puncture (LP) revealed 259 white blood cells (WBC), with 92% lymphocytes. The protein was 214 mg/dL, and glucose was 28 mg/dL. She was started on vancomycin, ceftriaxone, and metronidazole empirically. Over the next 7 days, her condition deteriorated. She was initially ambulating and conversational but became progressively more somnolent and inactive. Cerebrospinal fluid (CSF) cultures were negative, and antibiotics were discontinued. Other testing revealed negative antinuclear antibody (ANA), interferon‐gamma release assay (IGRA) for *Mycobacterium tuberculosis*, respiratory pathogens polymerase chain reaction (PCR) panel, urine culture, meningitis panel, CSF *Coccidioides* antibodies, and blood cultures. Because the outside evaluation was unrevealing and she was worsening, she was transferred to a higher level of care at our facility, Cook Children’s Medical Center (CCMC) in Fort Worth, Texas.

Physical examination on admission to our facility revealed a somnolent but responsive female with stable vital signs whose examination was otherwise unremarkable.

Her exposure history included a family trip to Mexico for 1 week in the preceding 6 months. The family denied any ingestion of undercooked meats or unpasteurized foods. They reported owning dogs with known flea and tick infestations 2 months before admission. She had no known flea or tick bites. She had no exposure to anyone with tuberculosis, SARS‐CoV‐2 infection, or other illnesses.

The initial differential diagnoses on arrival included autoimmune encephalitis and posterior reversible encephalopathy syndrome (PRES). Neurology, hematology, nephrology, infectious diseases, and intensive care unit (ICU) were all consulted to care for the patient.

Further evaluation included CSF testing for cytomegalovirus (CMV) PCR, Epstein–Barr virus (EBV) PCR, enterovirus PCR, herpes simplex virus (HSV) PCR, Mycoplasma PCR, and varicella zoster virus (VZV) PCR. Serum testing included antibodies for *Bartonella*, West Nile virus (WNV), and *Toxoplasma*. An IGRA for *Mycobacterium tuberculosis* was repeated. She was also evaluated for autoimmune disease with an autoimmune encephalitis panel, cytokines, an angiotensin‐converting enzyme level (ACE), and oligoclonal bands. A hemophagocytic lymphohistiocytosis (HLH) workup was undertaken and was negative. Additionally, bone marrow aspirate and biopsy were performed for pathology and cultures with negative results.

An initial MRI of the brain at our facility revealed progression of signal changes and enhancement in the deep gray nuclei, dorsal medulla, and area postrema. The initial location of findings favored an autoimmune process with infectious etiology considered unlikely.

On hospital Day 1 at CCMC, she was started on empiric methylprednisolone for presumed autoimmune encephalitis and progressive worsening. Her labs were no longer significant for leukopenia, with a WBC count of 6.98. Repeat imaging 2 days later demonstrated stable ventriculomegaly with a new hemorrhage in the left caudate nucleus with extension to the left lateral ventricle.

On examination, the patient continued to become less responsive, and on hospital day number three she developed respiratory failure requiring intubation. Two days after initiating steroids, there was no improvement, and plasmapheresis was initiated.

Follow‐up imaging showed continued abnormal signal changes in the bilateral caudate nuclei, thalami, and dorsal lower brainstem with intraventricular extension of hemorrhage and ventriculomegaly. CSF was obtained, and pan PCR for bacteria, fungi, and acid‐fast bacilli (AFB) was ordered. CSF revealed 209 WBC with a differential including 86% lymphocytes. The protein was 301 mg/dL, and glucose was 54 mg/dL. Antituberculous medications were added based on CSF findings of pleocytosis with lymphocytic predominance and elevated protein.

With no response to treatment and laboratory results unrevealing, a pan amoebae PCR test was sent to the Mayo Laboratories for *Naegleria fowleri*, *Balamuthia mandrillaris*, and *Acanthamoeba* species. The patient was started on fluconazole, pentamidine, miltefosine, and azithromycin empirically while awaiting PCR testing on hospital Day 10. Approximately 72 h later, the free‐living Amoebae PCR results revealed *B. mandrillaris.* Flucytosine and sulfadiazine were added 3 days later based on positive PCR results and in consultation with the CDC.

Continued MRI brain imaging on hospital Days 13 and 14 depicted disease progression through worsening inflammation and hemorrhagic encephalitis involving regions surrounding the ventricular system with diffuse sulcal effacement and implication of impending herniation, with permanent injury to the brainstem (Figure 1). MRI brain imaging alone was not sufficient to diagnose *B. mandrillaris* etiology; however, it was indicative of an atypical encephalitis.

**FIGURE 1:**
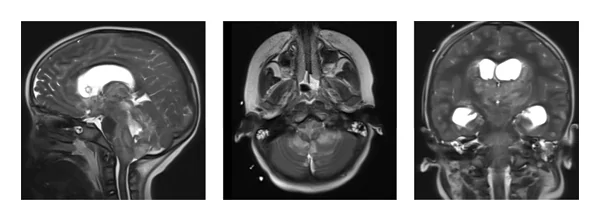
Left image: T2 sagittal, middle image: T2 axial, right image: SWI. Images: MRI brain W/WO hospital Day #13. MRI brain T2 depicts worsening central hemorrhagic amoebic encephalitis involving regions adjacent to the ventricular system with sites of intraparenchymal signal change and restricted diffusion. Inflammation intruded upon patient’s brainstem, leading to neurological devastation.

Unfortunately, the patient continued to deteriorate over the next few days with hemodynamic instability and met brain death criteria on hospital Day 16. After consulting the family, she was withdrawn from support.

## 3. Discussion

The diagnosis of *B. mandrillaris* encephalitis is challenging, and it requires a high index of suspicion [1]. The clinical features of the disease are nonspecific and may mimic other conditions such as bacterial or viral meningitis, encephalitis, or brain abscess [6]. Therefore, a detailed patient history and physical examination are crucial for identifying potential risk factors.

Recommendations for treatment of *B. mandrillaris* are quite variable and include pentamidine, flucytocine, sulfadiazine, a mold‐active azole, azithromycin, clarithromycin, and miltefosine. Definitive treatment has not been established because the disease is most often fatal, and the appropriate duration of therapy for survivors is unknown. Treatment decisions should be made in consultation with experts at the CDC.

Imaging studies such as MRI scans may reveal characteristic findings such as multiple brain lesions with ring enhancement, cerebral edema, and hydrocephalus [7]. However, our patient’s MRI findings were atypical in comparison to reported cases due to lacking ring‐enhancing lesions, location of signal changes, and lack of meningeal enhancement. The extensive hemorrhages primarily in regions surrounding the ventricular systems with the deep gray and brainstem involvement have not been extensively previously reported.

There are no specific lab findings that detect *B. mandrillaris*; however, certain lab findings such as pleocytosis, elevated CSF protein, and CSF cultures can raise clinical suspicion. Especially in immunocompetent patients such as ours, WBC counts remain unrevealing.

Taking a biopsy from the affected tissue or CSF examination can provide definitive evidence of infection [8]. However, obtaining a tissue biopsy is not always practical, especially in the case of brain biopsy, which has a high risk of complications. A more straightforward diagnostic approach is performing PCR on CSF or tissue samples to detect the amoebae’s genetic material [9]. In addition to traditional laboratory methods, new diagnostic techniques, such as metagenomics, and next‐generation sequencing (NGS) can detect and identify amoebae species in CSF, brain tissue, or other body fluids [10].

In conclusion, our patient’s clinical symptoms were consistent with reported characteristics of *B. mandrillaris* infections, such as: headache, fever, nausea, vomiting, altered mental status, and intracranial edema. However, these symptoms are not specific enough to confidently suspect amoebic encephalitis. Additionally, our patient’s neuroimaging did not characteristically include ring enhancing lesions or granulomas, which made the diagnosis more challenging. Early detection and clinical suspicion may have led to earlier pharmacological intervention. However, it remains difficult to conclude how early intervention needed to have been started to significantly improve our patient’s clinical course and outcome.

*B. mandrillaris* encephalitis is an uncommon but deadly infection that poses significant diagnostic challenges. Early recognition through clinical suspicion, appropriate imaging, and laboratory testing is critical for timely treatment, improving the patient’s outcome, and preventing further disease progression. PCR testing, metagenomics, and next‐generation sequencing have revolutionized amoebic infection research, offering more accurate diagnostic tools and valuable insights into the genetic composition and diversity of microorganisms. Their application in *B. mandrillaris* infection studies has the potential to improve diagnosis, identify co‐infections, and deepen our understanding of the amoeba’s pathogenicity, ultimately facilitating the development of effective treatment and prevention strategies.

## Funding

No funding was received for the preparation of this manuscript.

## Conflicts of Interest

The authors declare no conflicts of interest.

## Data Availability

The data that support the findings of this study are available on request from the corresponding authors. The data are not publicly available due to privacy or ethical restrictions.
